# Therapeutic effects of human amniotic mesenchymal stem cell-derived exosomes on stem cell proliferation in irradiated salivary glands via the Wnt pathway

**DOI:** 10.1515/biol-2025-1277

**Published:** 2026-02-18

**Authors:** Zhuang-Zhuang Li, Min Zhang, Xue Wang, Hui-Zhong Qi, Yue-Yue Wang, Jia-Yu Cao, Gui-Lin Huang, Ni-Ni Zhang

**Affiliations:** Department of Oral Maxillofacial Surgery, School and Hospital of Stomatology, Zunyi Medical University, Zunyi, 563003, China; Zunyi Medical University, Zunyi, 563006, China; Department of General Dentistry, School and Hospital of Stomatology, Zunyi Medical University, Zunyi, 563003, China; Department of Stomatology, The Fifth Affiliated (Zhuhai) Hospital of Zunyi Medical University, Zhuhai, 519180, China

**Keywords:** cell proliferation, human amniotic mesenchymal stem cell-derived exosomes, stem cells, submandibular gland radiation injury, Wnt signaling pathway

## Abstract

This study investigated the therapeutic potential of exosomes derived from human amniotic mesenchymal stem cells (hAMSCs-EXO) in enhancing stem cell proliferation and tissue regeneration in salivary glands following radiation-induced injury. 60 rats were randomly assigned to five experimental groups: (1) IR (irradiation followed by phosphate-buffered saline (PBS) injection), (2) XAV 939 (irradiation with subsequent administration of the Wnt signaling pathway inhibitor XAV-939), (3) XAV 939 + EXO (irradiation followed by hAMSCs-EXO and XAV-939), (4) EXO (irradiation followed by hAMSCs-EXO), and (5) Control (non-irradiated with PBS administration). Injections were administered on day 1 post-irradiation. Assessments included serial body weight, histopathology (H&E staining), immunofluorescence, 5-ethynyl-2′-deoxyuridine (EdU) incorporation, stem cell marker expression, and proliferation analysis on days 1, 3, 7, and 14. The EXO group demonstrated notable preservation of salivary gland architecture and significantly increased expression of stem cell markers, relative to other irradiated groups, with marked effects observed by day 7. EdU and Ki67 immunostaining further indicated a significant enhancement in proliferative activity following hAMSCs-EXO treatment. In contrast, administration of XAV-939 alone was associated with reduced proliferation and diminished stem cell marker expression. These findings suggest that hAMSC-derived exosomes promote stem cell proliferation and structural restoration in irradiated salivary gland tissue, potentially through activation of the Wnt signaling pathway.

## 1 Introduction

Head and neck malignancies are among the most common forms of cancer globally, with radiation therapy remaining a cornerstone of definitive and adjuvant treatment strategies [1]. Despite its therapeutic efficacy against tumors, radiotherapy often results in significant collateral damage to adjacent healthy tissues. Although various interventions have been explored to mitigate radiation-induced dysfunction of the salivary glands, their clinical effectiveness remains limited. Consequently, the development of novel therapeutic strategies for managing salivary gland injury is of considerable importance.

Mesenchymal stem cells (MSCs), a category of adult stem cells with multipotent differentiation potential, can be isolated from multiple tissue sources, including bone marrow, umbilical cord, and placenta. Among these, human amniotic mesenchymal stem cells (hAMSCs) are notable for their broad availability, non-invasive procurement, robust proliferative and differentiation capacities, and low immunogenicity. Additionally, hAMSCs exhibit multiple biological properties, including the promotion of angiogenesis, enhancement of cell survival, and modulation of immune responses, making them promising candidates for a wide range of therapeutic applications [2].

Exosomes (EXO) are nano-sized extracellular vesicles (30–150 nm in diameter) that play a key role in intercellular communication within the paracrine signaling system. They are secreted by various cell types and are present in multiple biological fluids, including blood, saliva, and cerebrospinal fluid. EXO release occurs under both physiological and pathological conditions. Previous studies have demonstrated that human amniotic mesenchymal stem cell-derived exosomes (hAMSCs-EXO) can enhance the migration and proliferation of skin fibroblasts, thereby facilitating wound healing [3], 4].

The Wnt signaling pathway, regulated by a family of evolutionarily conserved glycoproteins, plays a fundamental role in the maintenance of epithelial stem cells. In adult tissues, Wnt signaling is essential for preserving the integrity of the stem cell niche by promoting the proliferation and self-renewal of tissue-resident stem cells [5].

To date, no studies have specifically investigated the effects of hAMSCs-EXO on the proliferation of quiescent stem cells within radiation-damaged submandibular gland tissue. This study aimed to determine whether hAMSCs-EXO promote stem cell proliferation in salivary glands following radiation-induced injury through activation of the Wnt signaling pathway, thereby offering a potential therapeutic strategy for managing radiation-induced salivary gland dysfunction.

## 2 Materials and methods

### 2.1 Materials

The use of human amniotic membrane and animal tissues in this study was approved by the Institutional Ethics Committee. Human amniotic membrane samples were collected following cesarean section procedures performed in the Department of Obstetrics and Gynecology at the Affiliated Hospital of Zunyi Medical University. All donors were full-term pregnant women who had undergone prenatal screening for infectious diseases, with negative results. Informed consent was obtained from all participants and their families, and signed consent forms were collected.

Male Sprague Dawley (SD) rats used in the experimental procedures were procured from Hunan SJA Laboratory Animal Co., Ltd., under certificate number SCXK (Xiang) 2019-0004.

**Informed consent:** Informed consent has been obtained from all individuals included in this study.

**Ethical approval:** The research related to human use has been complied with all the relevant national regulations, institutional policies and in accordance with the tenets of the Helsinki Declaration, and has been approved by the Medical Ethics Committee of the Affiliated Stomatological Hospital of Zunyi Medical University (YJSKTLS-2019-2021-020H).

**Ethical approval:** The research related to animal use has been complied with all the relevant national regulations and institutional policies for the care and use of animals, and has been approved by the Medical Ethics Committee of the Affiliated Stomatological Hospital of Zunyi Medical University (YJSKTLS-2019-2021-020A).

### 2.2 Primary reagents and instruments

Cluster of differentiation (CD) antibodies including CD73-phycoerythrin (PE), CD90-PE, CD105-PE, and a cocktail of CD19/34/45/11b/Human Leukocyte Antigen-DR (HLA-DR) were procured from Fitzgerald. Anti-CD63 and anti-tumor susceptibility gene 101 (TSG101) antibodies (catalog numbers HY-P86367 and HY-P80924, respectively) were sourced from Med Chem Express (MCE). The horseradish peroxidase (HRP)-conjugated anti-rabbit secondary antibody (PR30009) was obtained from Proteintech. Dulbecco’s Modified Eagle Medium/Nutrient Mixture F-12 (DMEM-F12) and fetal bovine serum (FBS; catalog numbers 12634010 and 16000-044) were supplied by Gibco.

Anti-CD117 (c-Kit) and anti-Keratin 14 (K14) antibodies (catalog numbers 347321 and R381239, respectively) were purchased from Chengdu ZEN Bioscience Co., Ltd. The anti-SCA1 antibody (ab124688) was obtained from Abcam, and the anti-Ki67 antibody (GB151142) was sourced from Wuhan Servicebio Technology Co., Ltd.

Reagent kits included a dual-labeled multiplex immunofluorescence kit (AFIHC024) and a polymer-HRP universal anti-mouse/rabbit secondary antibody kit (AFIHC001), both from Suzhou Industrial Park Aifang Biopharmaceutical Tech Co., Ltd. 5-ethynyl-2′-deoxyuridine (EdU; HY-118411) was purchased from MCE, and the Click-iT EdU-488 cell proliferation detection kit (G1601) was sourced from Wuhan Servicebio Technology Co., Ltd. The Wnt signaling pathway inhibitor XAV 939 (M1796) was obtained from AbMole.

Instruments used in this study included a flow cytometer and a high-speed refrigerated centrifuge (both from Beckman, USA), a transmission electron microscope (Hitachi, Japan), a gel imaging system (BIO-RAD, USA), and a nanoparticle tracking analyzer (NanoSight, UK). Histological analyses were performed using an hematoxylin-eosin staining (H&E) staining kit (C0105S, Beyotime). Microscopy was conducted using a fluorescence inverted microscope and an inverted microscope (IX-71, Olympus, Japan).

### 2.3 Experimental methods

#### 2.3.1 Extraction and culture of hAMSCs

Placental amniotic membrane tissue was collected under sterile conditions and thoroughly rinsed with phosphate-buffered saline (PBS) containing 1 % double antibiotics. The tissue was finely minced and transferred to a 50 mL centrifuge tube, followed by the addition of 2–3 volumes of 0.05 % trypsin. Enzymatic digestion was performed under oscillation at 37 °C and 200 revolutions per minute (rpm) for 50 min.

Following the initial digestion, the tissue suspension was filtered through a 300-mesh stainless steel filter, rinsed with sterile balanced solution, and subjected to a second enzymatic digestion using a fresh enzyme solution. The initial filtrate was discarded, and the residual tissue retained on the filter was collected for further processing.

A digestion mixture comprising 1–2 volumes of type II collagenase and deoxyribonucleaseⅠ(DNase I) was added to the retained tissue, and oscillatory digestion was conducted at 37 °C and 200 rpm for 1 h. The resulting suspension was filtered again through a 300-mesh stainless steel filter, and an equal volume of complete culture medium was added to terminate enzymatic activity. The cell suspension was then centrifuged at 1,500 rpm for 7 min.

The resulting cell pellet, containing primary hAMSCs, was resuspended in complete medium and cultured under adherent conditions in a humidified incubator. Cell attachment was typically observed within 2–3 days. The culture medium was replaced every 2–3 days, and cells were subcultured until passage 3 (P3).

#### 2.3.2 Identification of hAMSCs

Third-passage hAMSCs were adjusted to a concentration of 5 × 10^5^ cells/mL, and 200 μL of the cell suspension was transferred into flow cytometry tubes. Fluorochrome-conjugated antibodies against human surface markers – CD73, CD90, and CD105 – as well as a mixed antibody panel targeting CD45, CD34, CD11b, CD19, and HLA-DR, were added under light-protected conditions. The samples were incubated for 25–30 min.

Following incubation, 2 mL of washing solution was added, and the tubes were centrifuged at 1,000 rpm. The supernatant was discarded, and the resulting cell pellets were resuspended in 500 μL of PBS for flow cytometric analysis.

In addition, third-passage cells were used to evaluate osteogenic and adipogenic differentiation potential.

#### 2.3.3 Separation and identification of hAMSCs-EXOs

FBS was ultracentrifuged at 120,000×*g* for 12 h to deplete endogenous EXOs, followed by filtration and storage at −20 °C for subsequent use. hAMSCs at passages three to five were cultured in medium supplemented with EXO-depleted serum for 48 h. The supernatant was then collected, and impurities were removed by sequential centrifugation at 800×*g* for 10 min and 2,000×*g* for 30 min at 4 °C, followed by filtration through a 0.22 μm membrane.

EXOs were isolated using a commercial EXO isolation reagent, following the manufacturer’s protocol. Briefly, 0.25 vol of extraction reagent were added to the pre-cleared supernatant, vortexed, and incubated overnight at 4 °C. The mixture was subsequently centrifuged at 10,000×*g* for 1 h at 4 °C. The supernatant was discarded, and the resulting pellet was resuspended and stored at −80 °C.

(1) Transmission electron microscopy (TEM) for morphological characterization

A 20–30 μL aliquot of the EXO suspension was applied to a copper grid and allowed to stand at room temperature for 1 min. Excess liquid was gently removed using filter paper. A 30 μL volume of 2 % phosphotungstic acid solution was then applied for negative staining and left at room temperature for 1 min. The solution was removed using filter paper, and the grid was air-dried. Morphological examination was performed using a transmission electron microscope, and representative images were acquired.

(2) Nanoparticle tracking analysis (NTA) for particle size and concentration

EXO samples were diluted with 1×PBS to an appropriate working concentration. The nanoparticle tracking analyzer was calibrated using 100 nm polystyrene microspheres. Measurements of particle size distribution and concentration were then performed and analyzed.

(3) Western blot analysis for surface marker identification

Protein was extracted from hAMSC-derived EXOs using radio-immunoprecipitation assay (RIPA) lysis buffer. The supernatant was collected, and total protein concentration was quantified using the bicinchoninic acid (BCA) assay. Following denaturation, proteins were separated by sodium dodecyl sulfate–polyacrylamide gel electrophoresis (SDS-PAGE) and transferred onto polyvinylidene fluoride (PVDF) membranes. After blocking with bovine serum albumin (BSA) for 2 h, membranes were incubated overnight at 4 °C with primary antibodies against cluster of differentiation 63 (CD63) and TSG101. Subsequent steps included washing with Tris-buffered saline containing Tween-20 (TBST), incubation with HRP-conjugated secondary antibodies in the dark, additional washes, and signal development for protein detection.

#### 2.3.4 Grouping and modeling of experimental animals

A total of 60 male SD rats, aged 6–8 weeks and of specific pathogen-free grade, were randomly assigned to five groups (*n* = 12 per group): IR group, XAV 939 group, XAV 939 + EXO group, EXO group, and Control group. Experimental assessments were conducted at four time points: days 1, 3, 7, and 14. The Control group underwent anesthesia without radiation exposure. A model of salivary gland radiation injury was established via targeted irradiation of the submandibular region.

For the irradiation procedure, animals in the four experimental groups were anesthetized via intraperitoneal injection of pentobarbital sodium (50 mg/kg), followed by exposure to an 18 Gy dose of radiation directed at the submandibular region, administered at a rate of 3 Gy/min. The remainder of the body was shielded using lead plates to limit off-target exposure.

Twenty-four hours post-irradiation, bilateral *in situ* injections into the submandibular glands were performed under anesthesia (50 mg/kg pentobarbital, intraperitoneally). The IR and Control groups received 100 μL of sterile PBS. The EXO and XAV 939 + EXO groups received 100 μL of PBS containing 200 μg of total exosomal protein. On the same day as EXO administration, the XAV 939 and XAV 939 + EXO groups received intraperitoneal injections of the Wnt signaling pathway inhibitor XAV-939 (1.25 mg/kg), administered four times per day.

Following irradiation, all animals were provided *ad libitum* access to food and water. Body weight was monitored regularly across all groups. At each designated time point (days 1, 3, 7, and 14), bilateral submandibular glands were harvested from three animals per group. Tissues were fixed in 4 % paraformaldehyde, embedded in paraffin, and sectioned for subsequent histological analyses. Immunofluorescence and hematoxylin and eosin (H&E) staining were performed to assess histopathological changes in the submandibular glands following EXO administration.

#### 2.3.5 H&E staining

Paraffin-embedded tissue sections were subjected to a standard H&E staining protocol. Sections were first deparaffinized and rehydrated, followed by immersion in hematoxylin solution for 5 min. After rinsing with water, differentiation and bluing steps were performed, followed by an additional wash.

Dehydration was carried out using graded ethanol solutions (85 % and 95 %), each for 5 min. Sections were then stained with eosin solution for 3 min. A second dehydration step was performed following eosin staining. Subsequently, sections were cleared in xylene and mounted using neutral balsam. Histological evaluation was performed under a light microscope.

#### 2.3.6 Immunofluorescence

Paraffin-embedded tissue sections were deparaffinized, followed by antigen retrieval, hydrogen peroxide treatment, and serum blocking. After completion of these preparatory steps, sections were incubated overnight at 4 °C with a primary antibody targeting Ki-67 antigen (Ki67) or CD117 (c-Kit), under controlled conditions. The corresponding fluorescent-conjugated secondary antibody was then applied, followed by microwave treatment to enhance signal intensity.

Subsequently, sections were incubated with a second primary antibody targeting Keratin 14 (K14) or Stem Cell Antigen-1 (SCA-1), followed by application of the appropriate fluorescent secondary antibody at room temperature.

Nuclear counterstaining was performed using 4′,6-diamidino-2-phenylindole (DAPI), and autofluorescence quenching was conducted for 5 min. Sections were then washed and mounted using an anti-fade mounting medium. Fluorescent signals were examined and imaged using a fluorescence microscope. Quantification of relative protein expression was performed by calculating mean fluorescence intensity across experimental groups (refer to Supplementary Materials).

#### 2.3.7 Immunofluorescence EdU detection

On day 7 following EXO administration, a single intraperitoneal injection of EdU was administered at a dose of 10 mg/kg. Four hours post-injection, submandibular gland tissues were harvested from all five experimental groups and processed into paraffin-embedded sections.

Following deparaffinization, 60 μL of permeabilization solution (0.5 % Triton X-100 in PBS) was applied to each section and incubated on a decolorization shaker for 10 min. Sections were then washed with PBS for 5 min. A total of 60 μL of EdU staining reaction solution was added to each section, followed by incubation in the dark at room temperature for 30 min.

Nuclear counterstaining was performed using DAPI solution. Sections were mounted with anti-fade mounting medium and analyzed by fluorescence microscopy.

### 2.4 Statistical methods

Statistical analyses and data visualization were performed using GraphPad Prism version 10.6. Quantitative data are expressed as mean ± standard deviation (mean ± SD). Comparisons between two independent groups were conducted using the unpaired *t*-test. For comparisons involving multiple groups, two-way analysis of variance was employed. A *p*-value of <0.05 was considered statistically significant.

## 3 Results

### 3.1 Identification of hAMSCs and hAMSCs-EXO

Flow cytometric analysis demonstrated that the isolated hAMSCs exhibited high expression of mesenchymal stem cell (MSC) surface markers, consistent with the established phenotypic profile of MSCs (Supplementary Figure 1). Additionally, the cells displayed osteogenic and adipogenic differentiation potential (Supplementary Figure 2).

**Figure 1:**
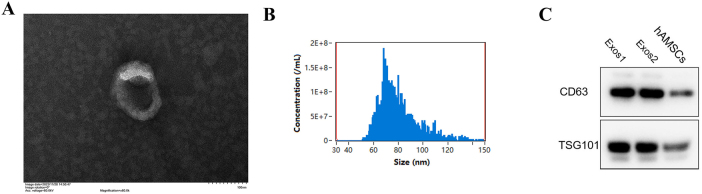
Identification of hAMSCs-EXO.

TEM revealed that hAMSCs-EXO exhibited round or near-round vesicular structures with bilayer membranes and a characteristic central depression, forming a typical “cup-shaped” morphology (Figure 1A). Nanoparticle tracking analysis showed that the average particle diameter of hAMSCs-EXO was approximately 80.0 nm (Figure 1B). Western blot analysis confirmed the presence of the exosomal surface markers CD63 and TSG101 (Figure 1C).

### 3.2 Restoration of salivary gland function in rats two weeks following EXO administration

#### 3.2.1 Changes in rat body weight

**Figure 2:**
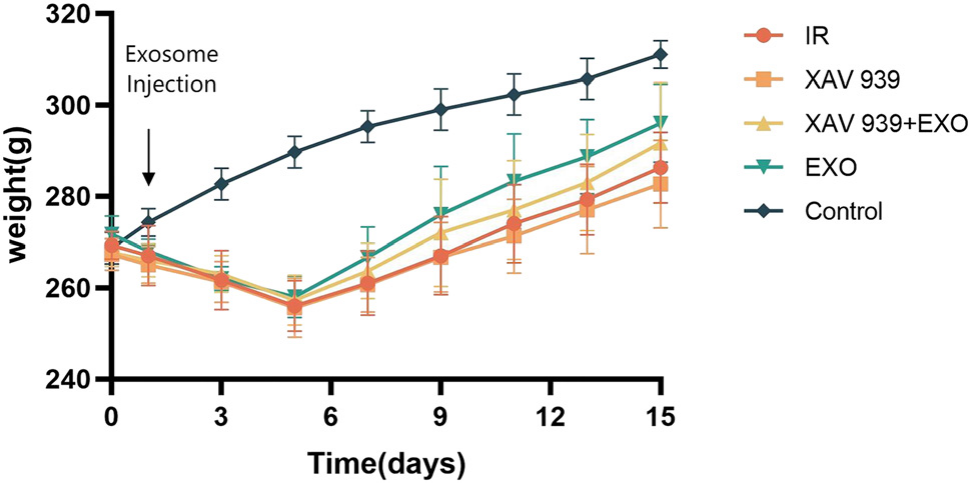
Body weight changes following EXO treatment over 14 days (*n* = 3 per group).

Except for the Control group, all irradiated groups exhibited a decline in body weight during the first five days following radiation-induced injury. Thereafter, body weight gradually recovered to near pre-radiation levels. Among the irradiated groups, the EXO group showed a more pronounced recovery in body weight compared to the IR, XAV 939, and XAV 939 + EXO groups, as illustrated in Figure 2.

#### 3.2.2 Morphological changes in rat submandibular gland tissue

Histological analysis revealed normal glandular architecture in the Control group, which did not undergo irradiation. In the EXO group, mild tissue atrophy was observed relative to the Control group; however, ductal structures remained largely intact, and the degree of tissue restoration was greater than that observed in the other irradiated groups.

**Figure 3:**
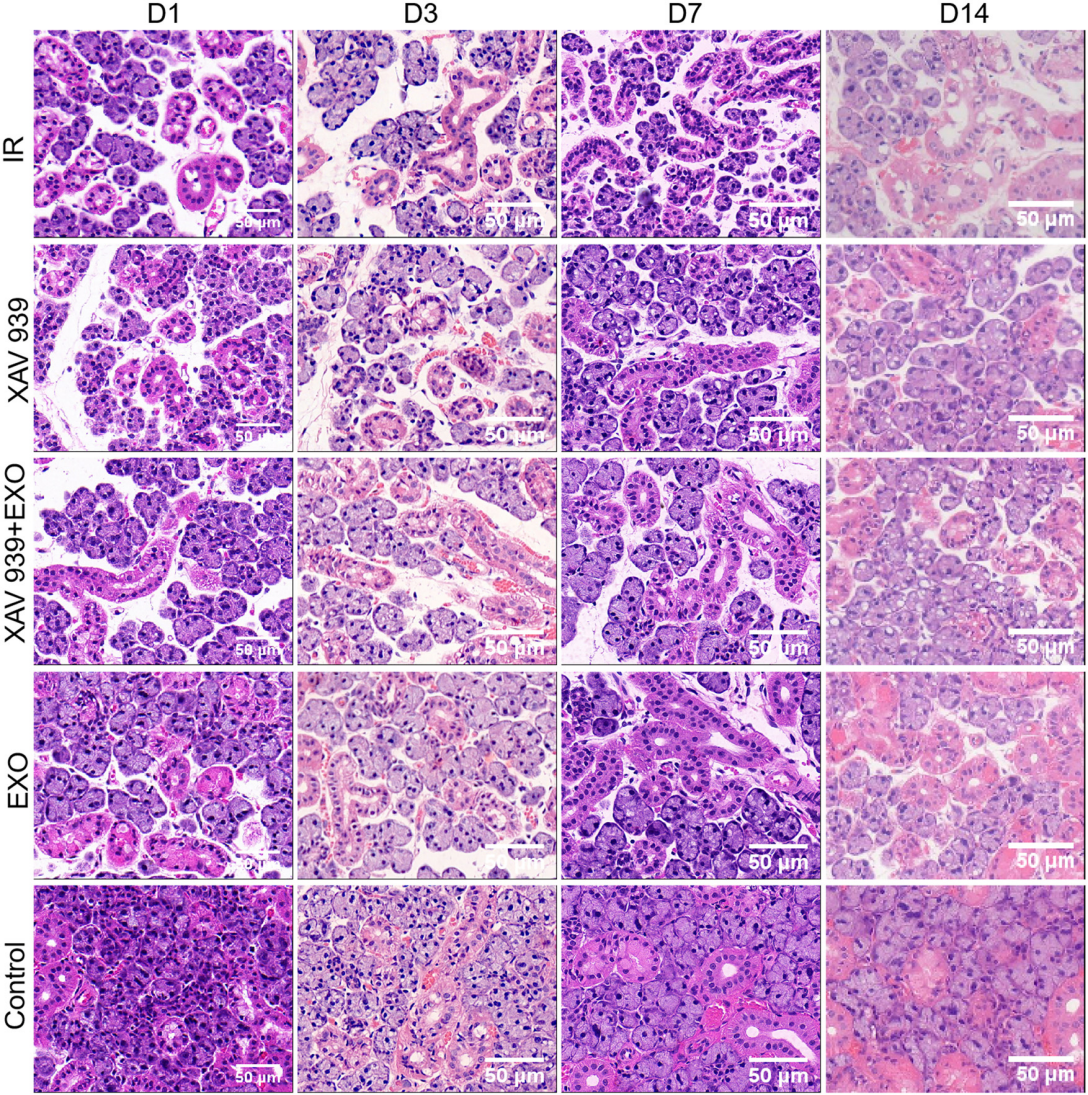
Histological changes in submandibular gland tissue after EXO injection in each group (*n* = 3).

Both the XAV 939 and XAV 939 + EXO groups exhibited similar histological features, demonstrating reduced regenerative capacity relative to the EXO group and comparable to the IR group. In the IR group, progressive tissue atrophy was evident over time, with marked degeneration of acinar and ductal structures, indicating limited tissue recovery (Figure 3).

**Figure 4:**
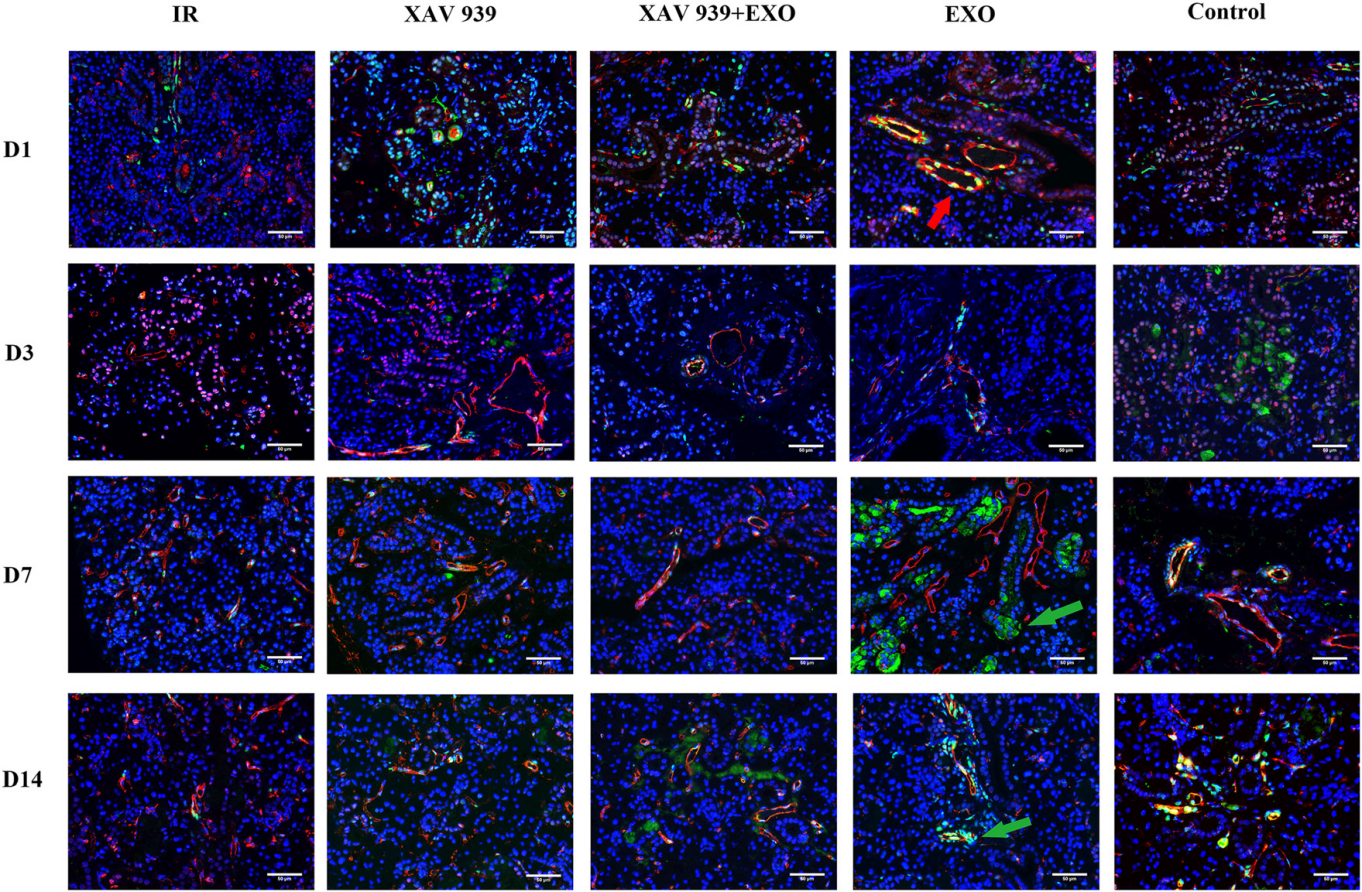
Immunofluorescence staining of c-Kit and SCA-1 in paraffin-embedded sections of rat submandibular gland tissue (*n* = 3). Images depict expression patterns on days 1, 3, 7, and 14 after EXO administration. The upper panels display immunofluorescence expression over time. Red arrows indicates co-localization of c-Kit and SCA-1; green arrows indicates strong c-Kit-positive expression. Scale bar: 50 μm.

#### 3.2.3 Immunofluorescence detection of rat submandibular glands

(1) Immunofluorescence staining for c-Kit (green) and SCA-1 (red) (Figure 4):

On day 1, weak co-expression of proto-oncogene c-Kit protein (c-Kit) and SCA-1 was observed around ductal structures in all five groups. By day 3, c-Kit-positive signals were present in the acinar cytoplasm of the Control group and in a small number of acini in the EXO group. On day 7, c-Kit expression was observed in the vasculature and ducts of the Control group and was strongly expressed in the acinar cytoplasm of the EXO group. No c-Kit signal was detected in the IR, XAV 939, or XAV 939 + EXO groups. By day 14, c-Kit expression surrounding SCA-1 in the EXO group had decreased. Minimal c-Kit positivity was detected in the XAV 939 + EXO group, and expression remained undetectable in the IR and XAV 939 groups.

For SCA-1, expression was noted in vessels and ducts on days 3 and 7, with no significant differences among the groups. On day 14, no upregulation of SCA-1 was observed in any group. However, co-localization of c-Kit and SCA-1 persisted in the vessels and ducts of the Control group.

**Figure 5:**
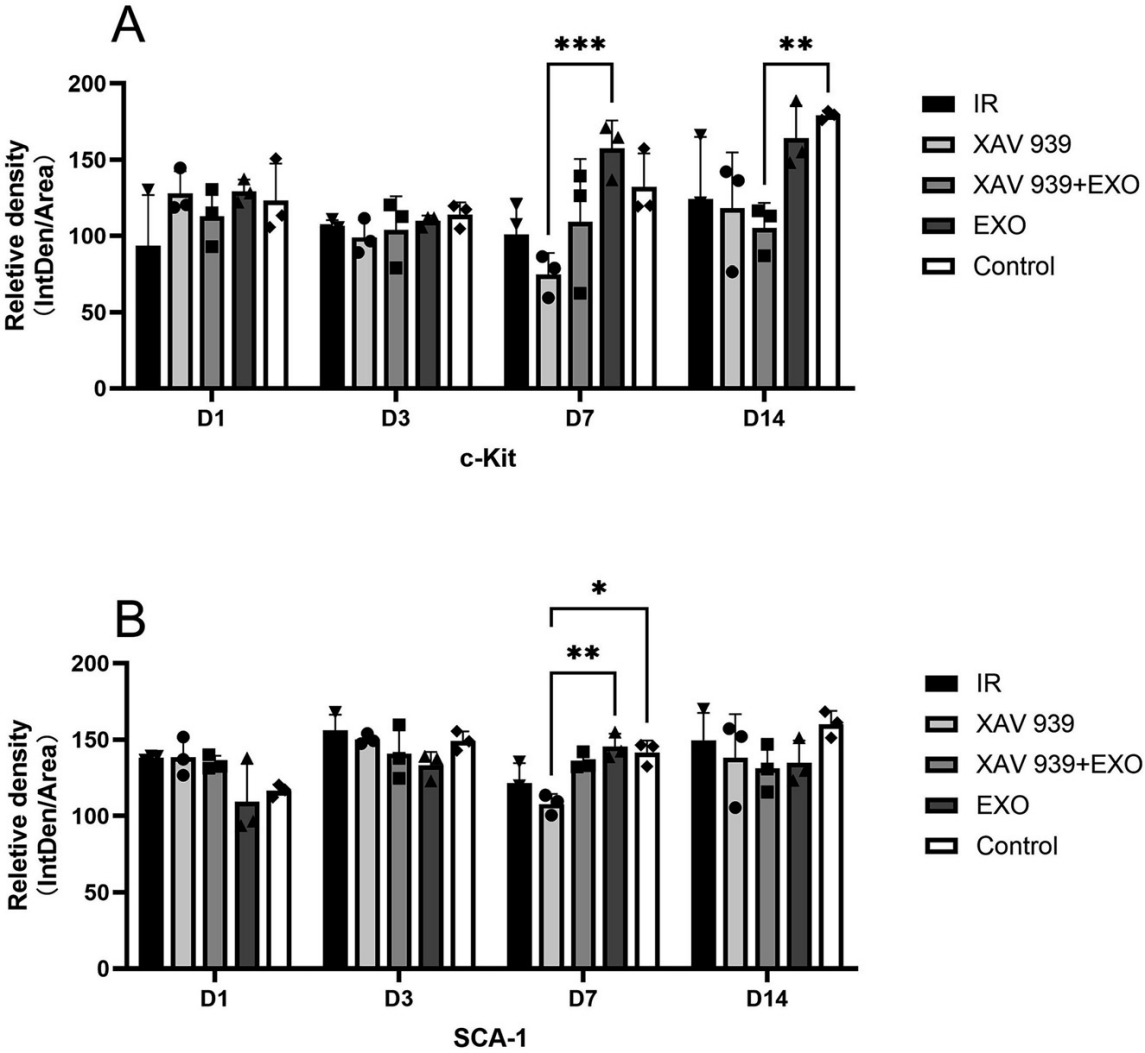
Quantitative fluorescence analysis of c-Kit and SCA-1 expression (*n* = 3). (A) Mean fluorescence intensity of c-Kit. (B) mean fluorescence intensity of SCA-1. *p < 0.05; **p < 0.01; ***p < 0.001.

Quantitative fluorescence analysis (Figure 5) showed that on day 7, c-Kit fluorescence intensity in the EXO group was significantly higher than in the other four groups, particularly greater than in the XAV 939 groups (*p* < 0.001). The lowest intensity was observed in the XAV 939 group. By day 14, c-Kit intensity in the EXO group was comparable to the Control group and remained higher than in the other three groups. The Control group also showed significantly higher c-Kit intensity than the XAV 939 + EXO group (*p* < 0.01). For SCA-1, a significant increase in fluorescence intensity was detected only on day 7 in the EXO group compared to the XAV 939 groups (*p* < 0.01); no significant differences were observed at other time points (*p* > 0.05).

**Figure 6:**
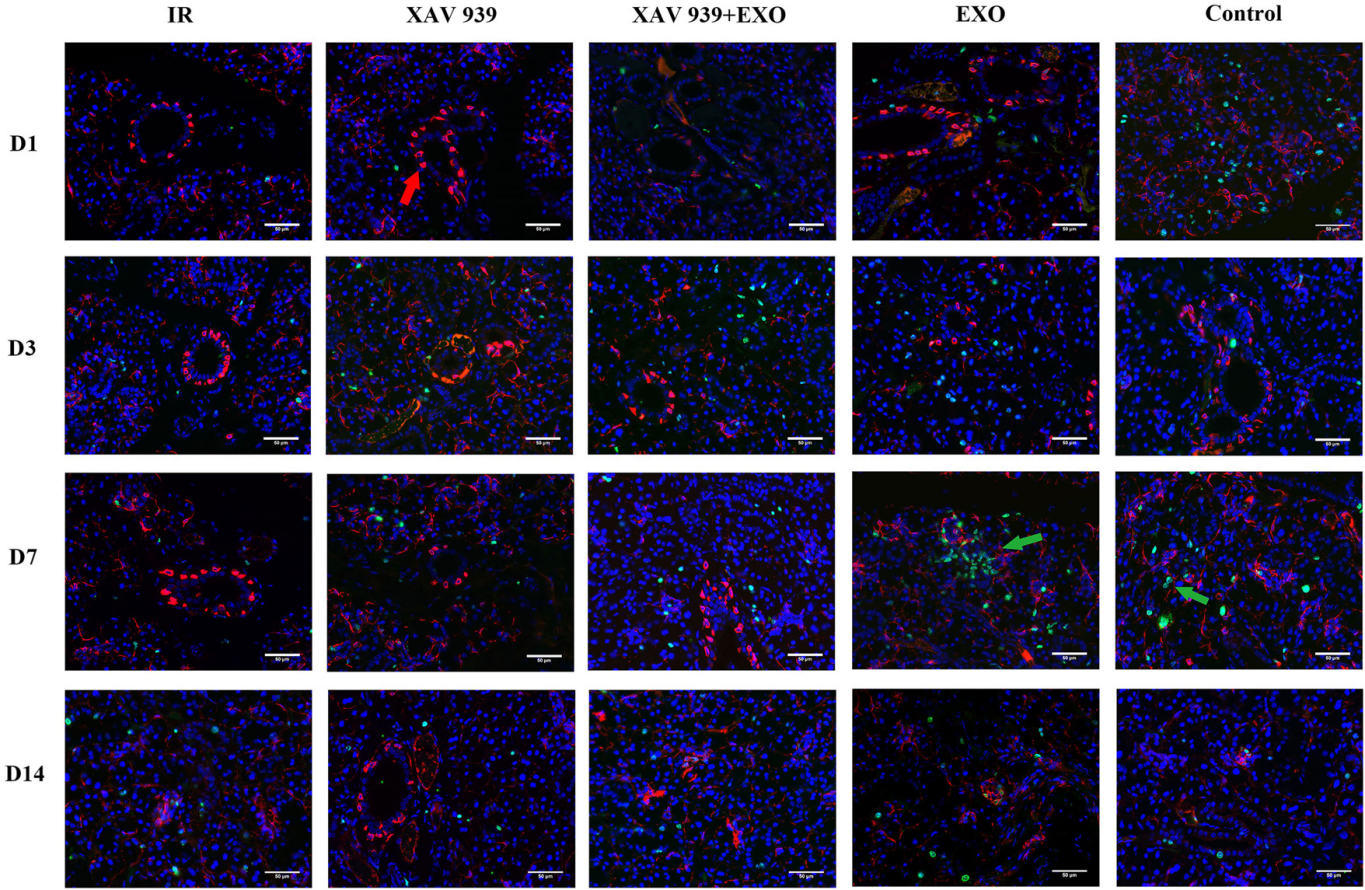
Immunofluorescence staining of Ki67 and K14 in paraffin sections of rat submandibular gland tissue (*n* = 3). Red arrows indicates K14 expression; green arrows indicates Ki67-positive expression. D7 control group green arrows denote mitotic figures. Scale bar: 50 μm.

(2) Immunofluorescence staining for Ki67 (green) and K14 (red) (Figure 6):

On day 1, strong nuclear expression of Ki67 was observed in the Control group, with weak expression detected in the other four groups. By day 3, Ki67 expression increased in both the EXO and XAV 939 + EXO groups, with visible mitotic figures in the EXO and Control groups. On day 7, the Control group continued to show Ki67-positive mitotic figures, and the EXO group exhibited strong, clustered Ki67 expression. In contrast, other groups demonstrated weak signals. By day 14, Ki67 staining appeared scattered across all groups, with no marked differences.

Regarding K14 expression, on day 1, the IR, XAV 939, and EXO groups exhibited expression primarily localized to the surface of thick ductal structures, while in the Control group, K14 was expressed on the surface of acinar cells. From days 3–14, no significant differences in K14 expression were observed among the five groups.

**Figure 7:**
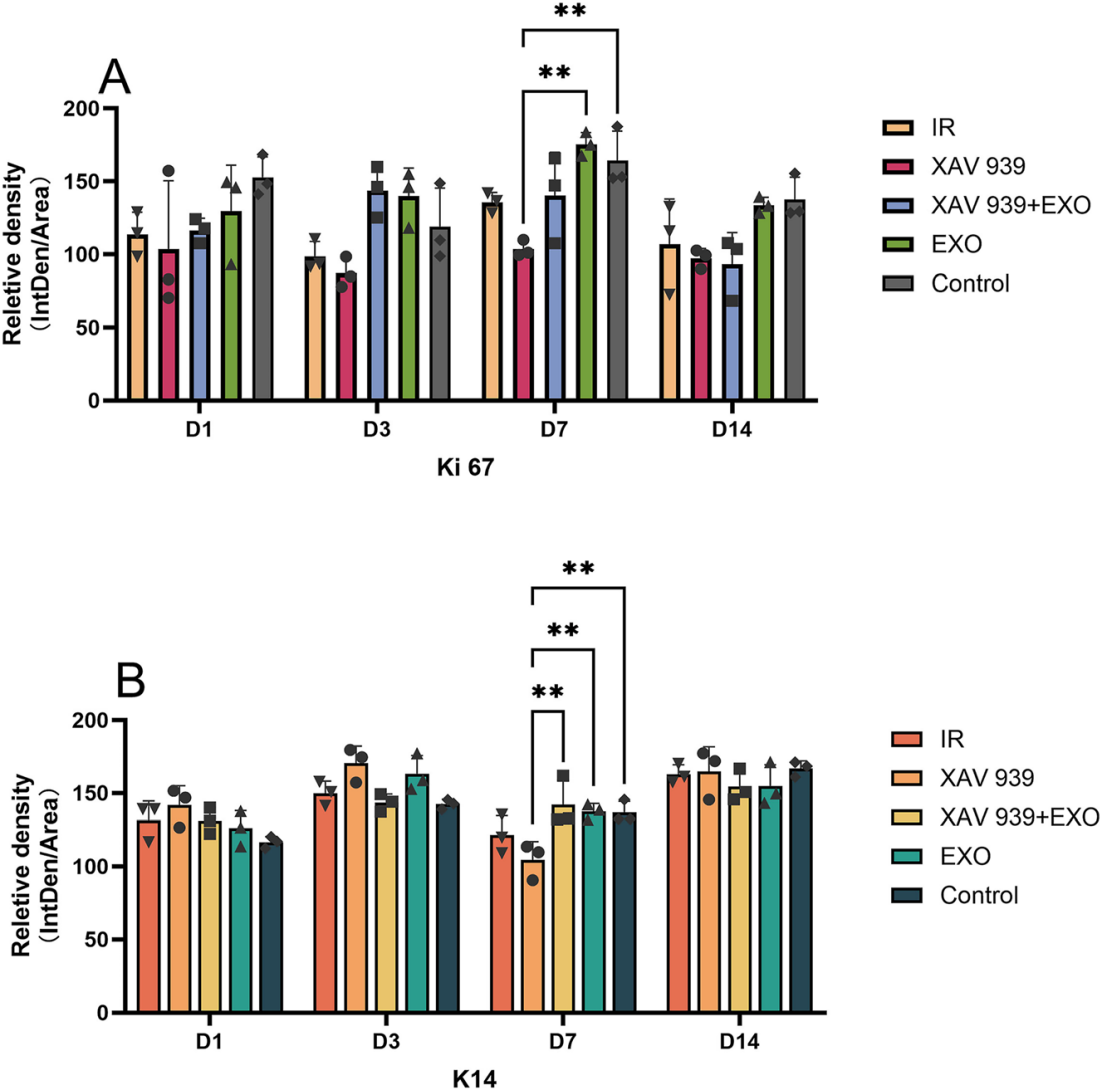
Quantitative fluorescence analysis of Ki67 and K14 expression (*n* = 3). (A) Mean fluorescence intensity of Ki67. (B) Mean fluorescence intensity of K14. **p < 0.01.

Quantitative fluorescence analysis (Figure 7) revealed that on day 7, Ki67 fluorescence intensity in the EXO group was significantly higher than in the other four groups, particularly higher than in the XAV 939 group (*p* < 0.01). The IR group also showed significantly higher intensity than the XAV 939 group (*p* > 0.05). On day 14, Ki67 intensity in the EXO group was comparable to the Control group and significantly higher than in the XAV 939 group (*p* > 0.05). Additionally, on day 7, K14 fluorescence intensity in the XAV 939 group was significantly lower than in the other three groups (*p* < 0.01).

**Figure 8:**
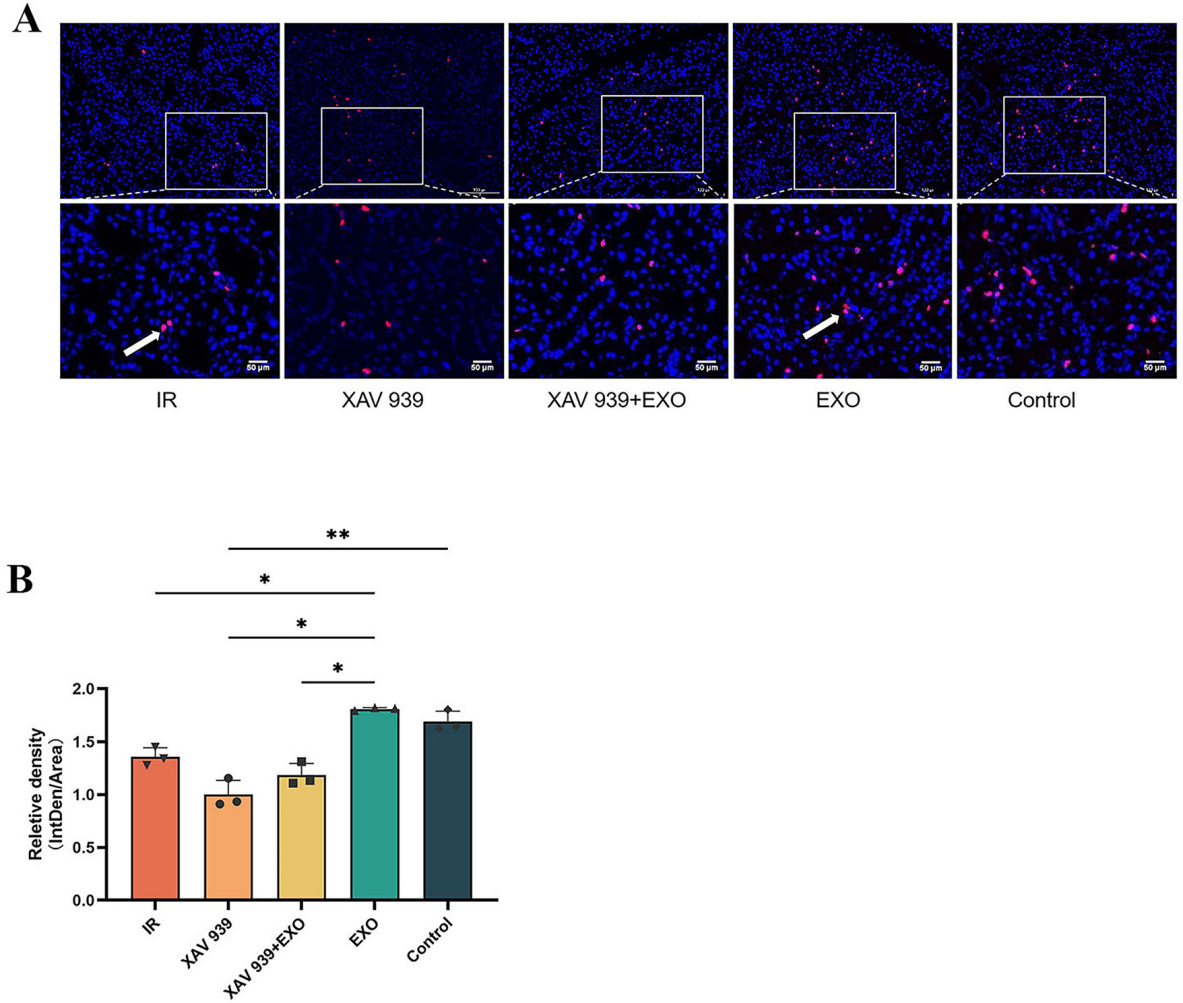
(A) EdU expression in paraffin sections of rat submandibular gland tissue. EdU-positive nuclei are indicated by red fluorescence. The lower panels show 4× magnification of the corresponding upper panels. White arrows indicate mitotic figures. Scale bar: 50 μm. (B) Quantitative fluorescence analysis of EdU expression (*n* = 3). *p < 0.05; **p < 0.01.

(3) EdU immunofluorescence staining (Figure 8A):

The EDU assay results (red fluorescence localized in the nucleus) showed that in the IR, XAV 939, and XAV 939 + EXO groups, EdU-positive cells were limited in number. In contrast, a marked increase in EdU-positive cells was observed in the EXO and Control groups, with visible mitotic figures present in both the IR and EXO groups.

Quantitative fluorescence analysis (Figure 8B) indicated that EdU intensity in the EXO group was slightly higher than in the Control group and significantly greater than in the IR, XAV 939, and XAV 939 + EXO groups (*p* < 0.05). The Control group also exhibited significantly higher EdU intensity than the XAV 939 group (*p* < 0.01).

## 4 Discussion

Radiation therapy for head and neck tumors frequently results in salivary gland injury, leading to complications such as hyposalivation and increased susceptibility to dental caries. Although advances in radiotherapy techniques and pharmacological interventions can alleviate some symptoms, strategies that promote fundamental repair of radiation-induced biological damage remain limited. Recent research has shifted toward EXO-mediated repair and tissue engineering, with hAMSCs-EXO demonstrating considerable therapeutic potential.

In the present study, rats exposed to radiation exhibited weight loss, reduced water intake, and lethargy by day 7 post-irradiation, consistent with clinical manifestations of salivary gland dysfunction [6], 7]. Although partial recovery was observed due to intrinsic repair mechanisms, body weight remained below normal levels [8]. Administration of hAMSCs-EXO alleviated these symptoms, suggesting an ability to promote endogenous stem cell proliferation and tissue regeneration.

Characterization of hAMSCs by flow cytometry confirmed the expression of MSC surface markers and demonstrated multipotent differentiation capacity. Isolated exosomes exhibited typical lipid bilayer “cup-shaped” morphology, with an average particle size of approximately 80 nm, and expressed membrane proteins such as CD63 and TSG101, consistent with established EXO characteristics [9].

Previous studies have shown that bone marrow mesenchymal stem cell-derived exosomes (BMMSC-EXO) [10] and adipose-derived mesenchymal stem cells-derived exosomes (ASC-EXO) [11] enhance wound healing and modulate fibroblast function. hAMSCs-EXO have also demonstrated therapeutic efficacy in promoting angiogenesis and diabetic wound healing [12], 13]. This study aimed to determine whether hAMSCs-EXO can promote stem cell proliferation and regeneration in radiation-damaged salivary glands.

Stem cell-based approaches are increasingly recognized as effective for repairing salivary gland injury caused by irradiation [14]. Among various stem cell markers, c-Kit and SCA-1 are commonly used to assess proliferation potential [15], 16]. Cells co-expressing both markers are believed to possess stem/progenitor cell properties [16]. In this study, strong c-Kit expression was observed in the acinar cytoplasm of the EXO group on day 7, while by day 14, c-Kit positivity appeared around SCA-1-expressing regions. This suggests that EXOs may promote the differentiation of stem cells into acinar (c-Kit^+^) and ductal/vascular (SCA-1^+^) lineages. Dormant stem cells were observed to co-express both markers.

Immunohistochemical studies have shown that c-Kit and SCA-1 expression is typically localized to intercalated, striated, and excretory ducts, but not to acini [17]. Another study [18] further confirmed that c-Kit^+^ cells in human salivary glands are located exclusively in excretory ducts. These findings are consistent with our observations. Quantitative analysis demonstrated that both c-Kit and SCA-1 expression levels were significantly higher in the EXO group compared to other groups. This effect was markedly suppressed in the presence of the Wnt pathway inhibitor XAV 939, indicating that EXOs may promote stem cell differentiation via activation of the Wnt signaling pathway.

Keratin 14 (K14), another epithelial marker, has been proposed to play a role in salivary gland epithelial differentiation and may serve as an indicator of progenitor cell populations such as intraflagellar transport 140 kDa protein/keratin 14 (IFT140^+^/K14^+^) cells [19]. Ki67 is a nuclear antigen associated with cell proliferation and has been used to assess the mitotic activity of submandibular gland stem cells [20]. While previous studies suggest that K14^+^ and c-Kit^+^ cells can self-renew and contribute to acinar regeneration [21], others have reported that K14 and c-Kit label distinct, non-overlapping cell populations and that K14^+^ ductal cells may lack regenerative capacity [22], 23].

In the present study, K14 expression was observed in both ductal and acinar cells but did not co-localize with Ki67. Furthermore, there were no significant differences in K14 expression among groups, suggesting that K14 may not be a reliable marker for salivary gland stem cells in this model. In contrast, Ki67 expression was significantly elevated in the EXO group on days 3 and 7, with clustered mitotic figures indicative of active proliferation. This proliferative effect was again suppressed by XAV 939, further supporting the involvement of Wnt signaling in EXO-mediated stem cell activation.

Overall, our findings indicate that hAMSC-derived EXOs promote the proliferation of c-Kit^+^ and SCA-1^+^ salivary gland stem cells via the Wnt signaling pathway but do not promote proliferation of K14^+^ ductal cells. Previous studies have shown that most salivary gland stem cells – representing distinct cell populations – reside within the acinar compartment and contribute to regeneration following injury [24]. Although no single definitive marker for salivary gland stem cells has been universally accepted [25], this study supports the utility of c-Kit and SCA-1 as reliable indicators.

Histological analysis by H&E staining confirmed that the EXO group exhibited improved preservation and regeneration of glandular architecture, with only mild atrophy, compared to other irradiated groups. In contrast, the XAV 939 group demonstrated a repair profile similar to the IR group, indicating that inhibition of Wnt signaling suppresses the regenerative effect of EXOs.

To further validate the role of EXOs in stimulating endogenous stem cell proliferation, EdU incorporation assays were performed. The EXO group exhibited abundant EdU-positive cells and mitotic figures, indicating robust deoxyribonucleic acid (DNA) synthesis and tissue regeneration. Quantitative analysis revealed significantly higher EdU fluorescence intensity in the EXO group compared to the IR, XAV 939, and XAV 939 + EXO groups (*p* < 0.05), reinforcing the conclusion that EXOs facilitate tissue repair through Wnt pathway activation.

Previous studies have identified sex-determining region Y-box 9 (Sox9^+^) cells as a progenitor population involved in salivary gland regeneration via the Wnt pathway [26]. Administration of XAV 939, a potent inhibitor of Wnt/β-catenin signaling, has been shown to exacerbate radiation-induced glandular damage, further underscoring the importance of Wnt signaling in tissue repair. Moreover, co-activation of the Wnt/β-catenin and TGF-β pathways has been reported to mitigate salivary gland injury during radiotherapy for head and neck tumors [27].

While hAMSCs-EXO exhibit clear therapeutic potential, the precise molecular mechanisms remain incompletely understood. Exosomes contain diverse biomolecules, including proteins, lipids, and microRNAs (miRNAs) that contribute to their biological activity [28]. For instance, exosomes derived from human umbilical cord MSCs are enriched in miRNAs that regulate cyclin-related genes and activate Wnt/β-catenin signaling, thereby promoting dermal cell proliferation and differentiation [29]. Similarly, exosomes from human urinary-derived stem cells have been shown to upregulate Wnt3a, GSK3β, and Axin expression in salivary gland tissues, indicating activation of the Wnt pathway [30]. These effects may be mediated by miRNAs that inhibit apoptosis and support the survival of functional stem/progenitor cells [31].

Building upon the findings of this study and previous research, we hypothesize that hAMSC-EXOs promote the proliferation and differentiation of endogenous stem cells in the submandibular glands by modulating Wnt-related signaling pathways. This mechanism likely contributes to enhanced regenerative and reparative capacity following radiation-induced injury. These findings suggest that EXOs may facilitate stem cell activation in irradiated salivary gland tissue through Wnt pathway activation.

Supporting this hypothesis, a previous study [32] demonstrated that delivery of microRNA-133b-3p (miR-133b-3p) can regulate disco-interacting protein 2 (DIP2)-mediated DNA methylation, thereby promoting the expansion of c-Kit^+^ progenitor cells within the apical bud of radiation-injured glandular epithelium. As exosomes are known to carry diverse bioactive molecules, including miRNAs, it is plausible that hAMSCs-EXO contain miR-133b-3p or similar regulatory elements. Further investigation is warranted to identify the specific molecular components responsible for Wnt pathway activation and stem cell proliferation. In addition to the Wnt/β-catenin pathway Wnt pathway), exosomes may also contribute to tissue repair by activating other canonical signaling cascades, such as Sonic Hedgehog (SHH), particularly within the acinar compartment [33].

Prior studies have also indicated that Sox9^+^ cells may function as a progenitor population involved in Wnt-mediated salivary gland regeneration [26]. Activation of intra-acinar SHH and WNT signaling has been shown to mitigate radiation-induced tissue damage [27]. In the present study, the use of XAV 939, a potent Wnt/β-catenin pathway inhibitor, exacerbated glandular injury, further supporting the role of Wnt signaling in endogenous tissue repair processes.

Taken together, the findings indicate that hAMSC-derived EXOs enhance stem cell proliferation in radiation-injured submandibular glands and that this effect is significantly attenuated upon inhibition of the Wnt pathway. These results support the conclusion that the reparative effects of EXOs are, at least in part, mediated by Wnt pathway activation in salivary gland stem cells.

Currently, there is no universally accepted set of surface markers for identifying salivary gland stem cells. This study supports the utility of c-Kit and SCA-1 as potential proliferation-associated markers, although further validation is required to confirm their specificity and reliability. It is important to note that the repair mechanisms following radiation-induced salivary gland injury are complex and involve multiple pathways beyond stem cell proliferation, including apoptosis, immune modulation, and neurovascular remodeling. The present study focused specifically on the proliferative response induced by hAMSCs-EXO and did not assess these additional factors.

Future studies will incorporate Wnt/β-catenin gene-deficient mouse models and stem cell lineage tracing techniques (e.g., tamoxifen-inducible systems) to further elucidate the mechanisms by which miRNAs contained in hAMSCs-EXO activate dormant stem cell populations. These approaches will provide deeper insight into the molecular regulation of salivary gland regeneration.

In summary, hAMSC-derived exosomes promote the proliferation of c-Kit^+^/SCA-1^+^ salivary gland stem cells via activation of the Wnt signaling pathway, independent of K14^+^ cell involvement. This regenerative mechanism offers experimental evidence supporting the development of targeted, exosome-based therapies for salivary gland repair. Such strategies may ultimately contribute to improved quality of life for patients undergoing radiotherapy for head and neck cancers.

## 5 Conclusions

Based on the experimental findings, hAMSCs-EXOs enhance the proliferation of endogenous stem cells in a radiation-induced salivary gland injury model in Sprague-Dawley rats (SD rats). The observed attenuation of this effect following Wnt pathway inhibition suggests that hAMSCs-EXOs promote stem cell proliferation, at least in part, through modulation of the Wnt signaling pathway.

## Supplementary Material

Supplementary Material

Supplementary Material

Supplementary Material
