# Supplementary material for: Therapeutic effects of human amniotic mesenchymal stem cell-derived exosomes on stem cell proliferation in irradiated salivary glands via the Wnt pathway
Source: Open Life Sci. 2026 Feb 18;21(1):20251277. doi: 10.1515/biol-2025-1277 (PMC12917546; doi:10.1515/biol-2025-1277)
Supplement: Supplementary file 1 — Supplementary Material [file j_biol-2025-1277_suppl_001.docx]

**Additional Experimental Methods**
Quantification of Immunofluorescence Analysis:

Immunofluorescence images were quantitatively analyzed using ImageJ software. For each group at each time point, the mean fluorescence intensity (MFI) was calculated from three independent samples (n = 3) to determine the relative expression levels of target proteins. Briefly, all images were preprocessed in a standardized manner by converting to 8-bit grayscale and applying a uniform threshold to subtract background signals. Regions of interest (ROIs) corresponding to individual cells were precisely delineated. Integrated density (IntDen) and area (Area) were measured in a batch-processing mode. The MFI for each cell was calculated using the formula: MFI = IntDen / Area. Data were exported for further analysis, and the mean values ± standard error of the mean (SEM) were calculated for each group at each time point.
