# Supplementary figures and images for: Therapeutic effects of human amniotic mesenchymal stem cell-derived exosomes on stem cell proliferation in irradiated salivary glands via the Wnt pathway
Source: Open Life Sci. 2026 Feb 18;21(1):20251277. doi: 10.1515/biol-2025-1277 (PMC12917546; doi:10.1515/biol-2025-1277)

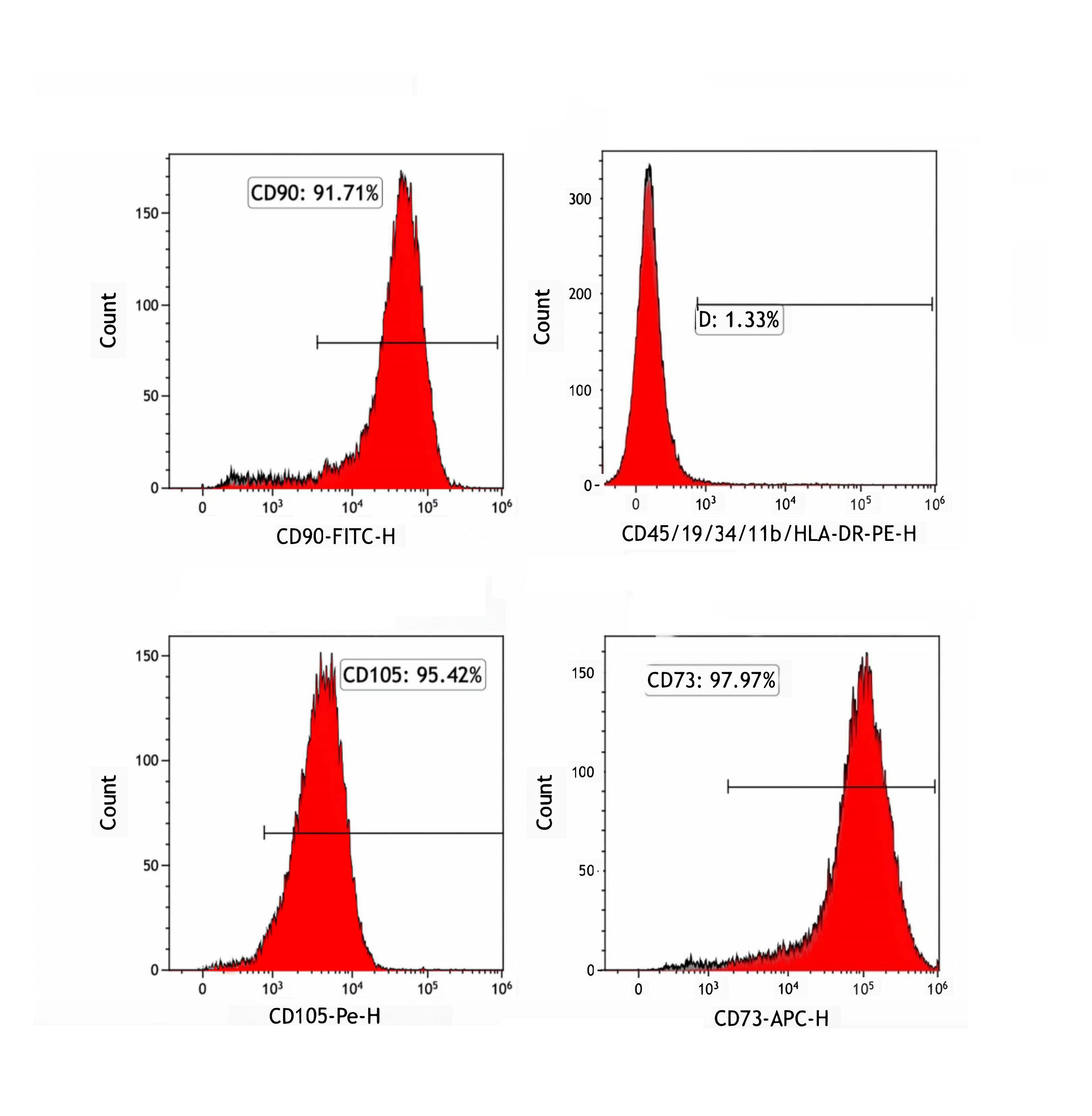

Supplement: Supplementary file 2 — Supplementary Material [file j_biol-2025-1277_suppl_002.zip › j_biol-2025-1277_suppl_002.tif]

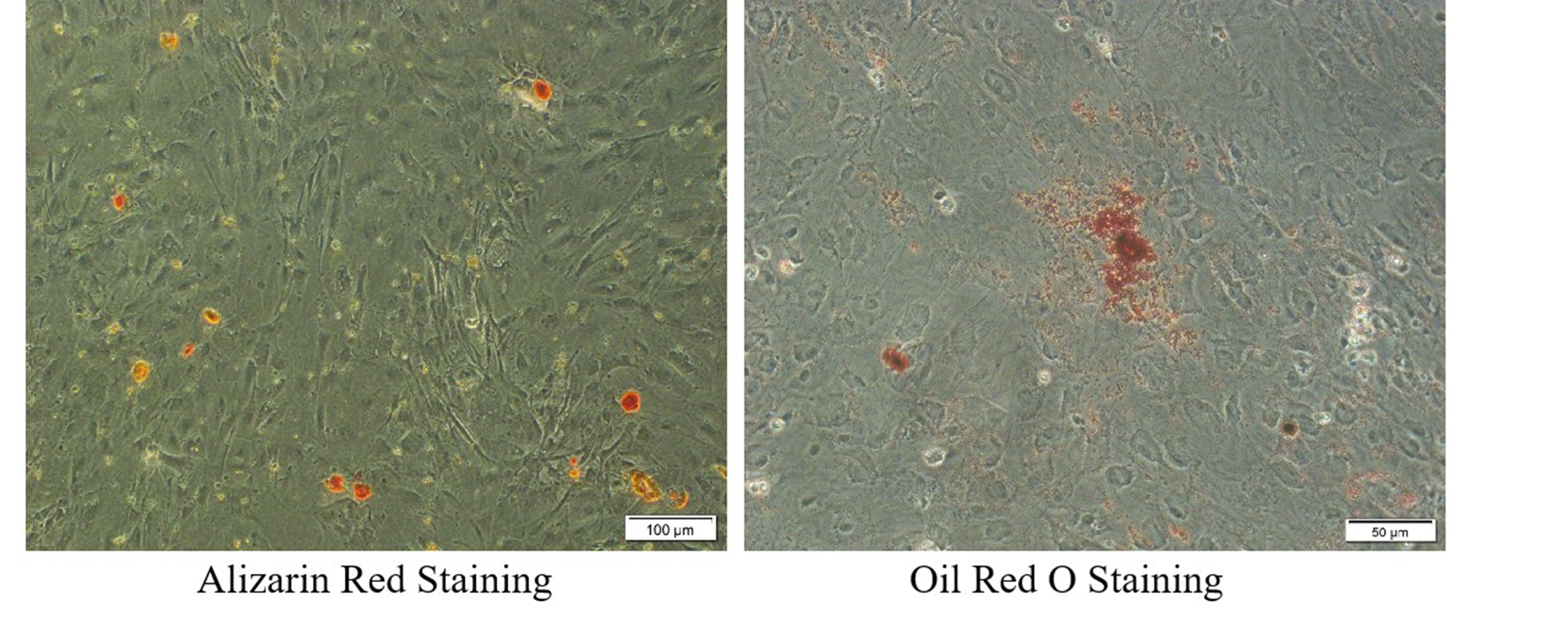

Supplement: Supplementary file 3 — Supplementary Material [file j_biol-2025-1277_suppl_003.zip › j_biol-2025-1277_suppl_003.tif]
